# Supplementary material for: Molecular architecture of a cylindrical self-assembly at human centrosomes
Source: Nat Commun. 2019 Mar 11;10:1151. doi: 10.1038/s41467-019-08838-2 (PMC6411776; doi:10.1038/s41467-019-08838-2)

Supplementary Information for

Molecular architecture of a cylindrical self-assembly at human centrosomes

Tae-Sung Kim, Liang Zhang, Jong Il Ahn, Lingjun Meng, Yang Chen, Eunhye Lee, Jeong Kyu Bang, Jung Mi Lim, Rodolfo Ghirlando, Lixin Fan, Yun-Xing Wang, Bo Yeon Kim, Jung-Eun Park, Kyung S. Lee

This file includes:

Supplementary Figures 1–9

Supplementary References

Supplementary Tables 1–5

Supplementary Figure 1

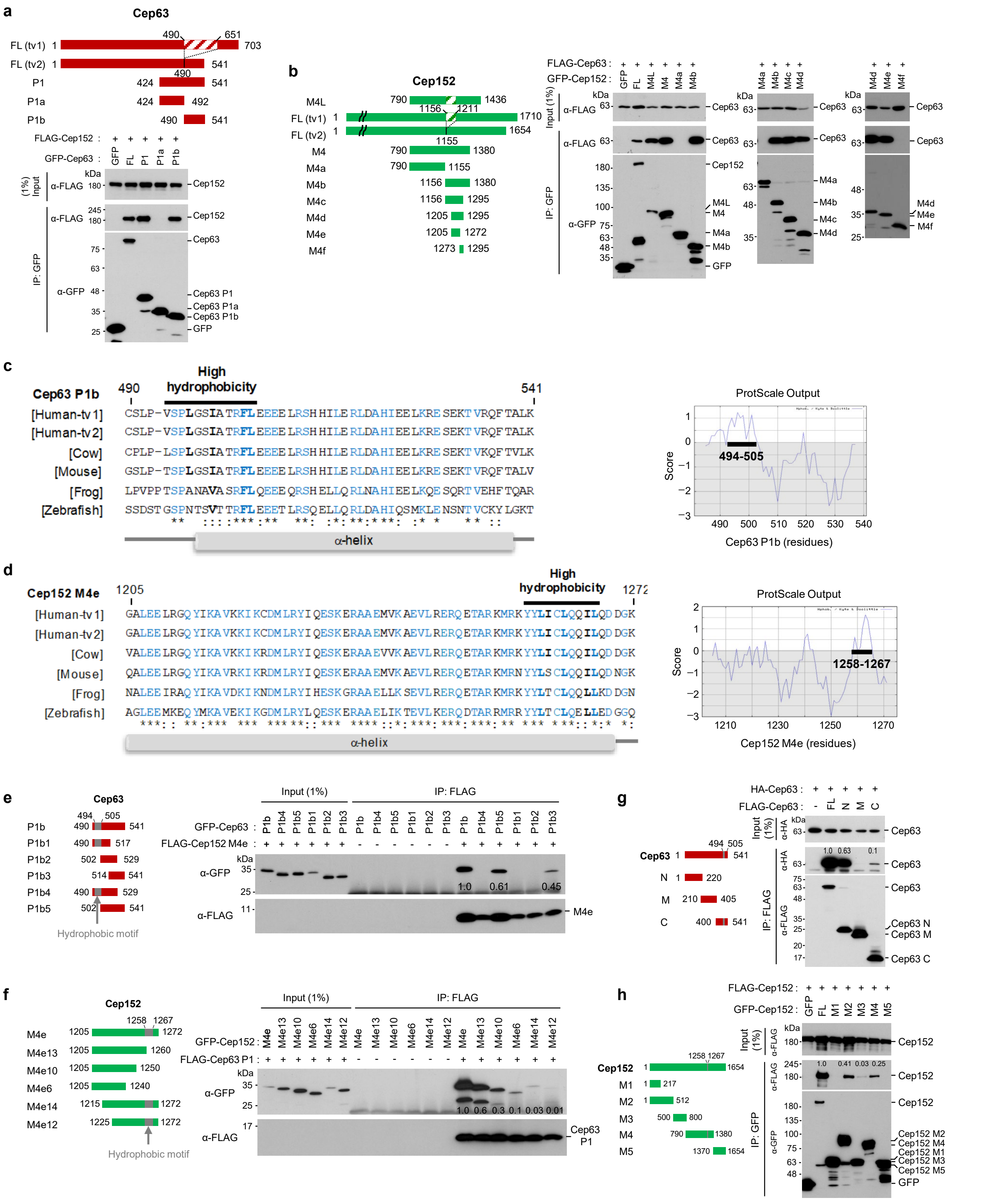

Supplementary Figure 1

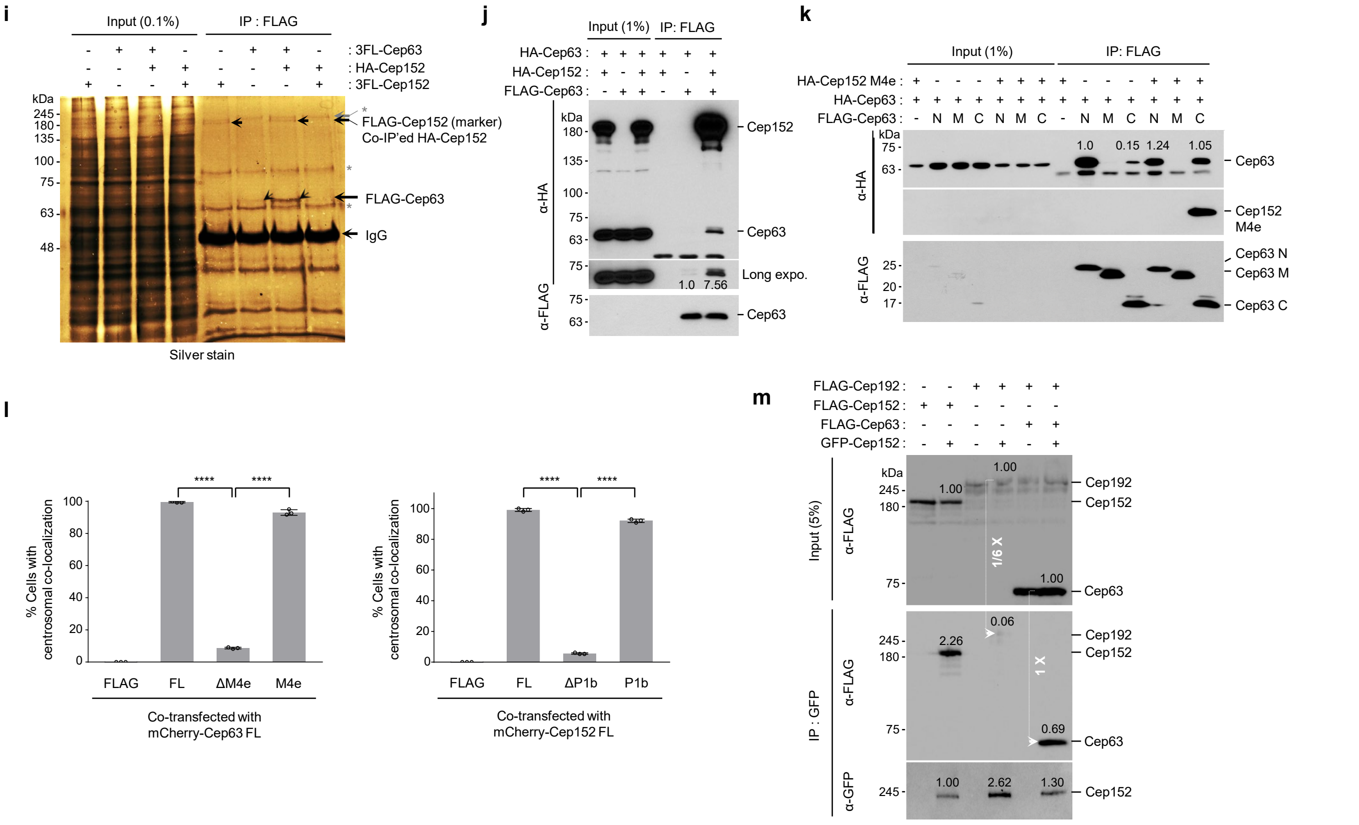

Supplementary Fig. 1. Identification of Cep63 and Cep152 domains required for their heteromeric and homomeric interactions. a, IP and immunoblotting analyses using transfected HEK293 cells. The schematic diagrams of Cep63 transcription variant (tv) 1 (GenBank accession number NP_079456.2) and tv2 (GenBank accession number NP_001035859.1) are shown. The hatched box denotes the region (residues 490–651) present only in Cep63 tv1. **b**, IP and immunoblotting analyses conducted similarly to (**a**) are shown. Cep152 tv1 (GenBank accession number: NP_001181927.1) contains a region (hatched box, residues 1156–1211) not present in the shorter Cep152 tv2 form (GenBank accession number: NP_055800.2). **c, d,** Multiple sequence alignment for human Cep63 P1b (residues 490–541) (**c**) or Cep152 M4e (residues 1205–1295) (**d**) were performed using the Clustal Omega software. The primary amino-acid sequences of Cep152 orthologs are: human Cep152 tv1 (NCBI RefSeq: NP_001181927.1), human Cep152 tv2 (NCBI RefSeq: NP_055800.2), cow Cep152 (UniProtKB: E1BCR3), mouse Cep152 (UniProtKB: A2AUM9), frog Cep152 (UniProtKB: Q498G2), and zebrafish Cep152 (UniProtKB: X1WBK2). The primary amino-acid sequences of Cep63 orthologs are: human Cep63 tv1 (NCBI RefSeq: NP_079456.2), human Cep63 tv2 (NCBI RefSeq: NP_001035859.1), cow Cep63 (UniProtKB: E1BDP9), mouse Cep63 (UniProtKB: Q3UPP8), frog Cep63 (UniProtKB: B9V5F5), and zebrafish Cep63 (UniProtKB: Q6PGZ0). Hydrophobic residues marked in bold-face type (L497, I500, F504, and L505 in Cep63 P1b (**c**), as well as L1260, I1261, L1263, I1266, and L1267 in Cep152 (**d**)) are mutated to Ala to generate 4A and 5A mutants, respectively, for further studies (see below). Blue, identical residues; gray bar, α-helix as predicted by the PSIPRED server. Hydrophobicity analysis using the ExPASy Kyte-Doolittle method [1] is shown on the right. **e**–**k**, IP and immunoblotting using transfected HEK293 cells. The gray boxes making residues 494–505 in (**e**) and (**g**) and residues 1258–1267 in (**f**) and (**h**) indicate the hydrophobic regions in Cep63 P1b and Cep152 M4e, respectively. Numbers, relative signal intensities normalized to the amounts of immunoprecipitated ligands. Asterisks in (**i**), cross-reacting proteins with anti-FLAG beads. Arrows, FLAG-Cep63 and HA-Cep152 proteins. Results in (**i**) show that FLAG-Cep63 efficiently coprecipitates HA-Cep152 from transfected cells. Note that accessing the *in vivo* binding stoichiometry between these two

proteins would be difficult because of the homomerization capacity of both Cep63 and Cep152, as shown Supplementary Fig. 1g, h, and their unknown levels of soluble forms present in transfected cell lysates. Also, note that the IP for (**j**) was performed using a mixture of lysates from cells transfected separately with each of the indicated constructs. Under these conditions, while FLAG-Cep63 coprecipitated HA-Cep63 weakly (likely because a large fraction of individually expressed Cep63 is already homomerized through its N-terminus, as shown in Supplementary Fig. 1g), the provision of HA-Cep152 greatly increased the level of coprecipitated HA-Cep63 through the capacity of Cep152 to mediate heteromeric interactions with Cep63 (see Fig. 3d for details). **l,** Quantification for the samples provided in Fig. 1e was conducted with three independent experiments (100 to 208 cells/sample/experiment). Error bars, standard deviation; ****, $P < 0.0001$ (unpaired two-tailed *t*-test). **m,** Immunoprecipitation (IP) and immunoblotting analyses using transfected HEK293 cells transfected with the indicated constructs. Numbers indicate relative expression levels in the input and amounts of coimmunoprecipitated target proteins normalized by the levels of immunoprecipitated ligands. Note that the level of the Cep192-Cep152 interaction is substantially weaker than the Cep63-Cep152 or Cep152-Cep152 interaction. In addition, earlier studies have shown that Cep192 and Cep152 localize to pericentriolar regions at least 40 nm away from each other [2,3], suggesting that their interaction, if present, may likely be limited.

Supplementary Figure 2

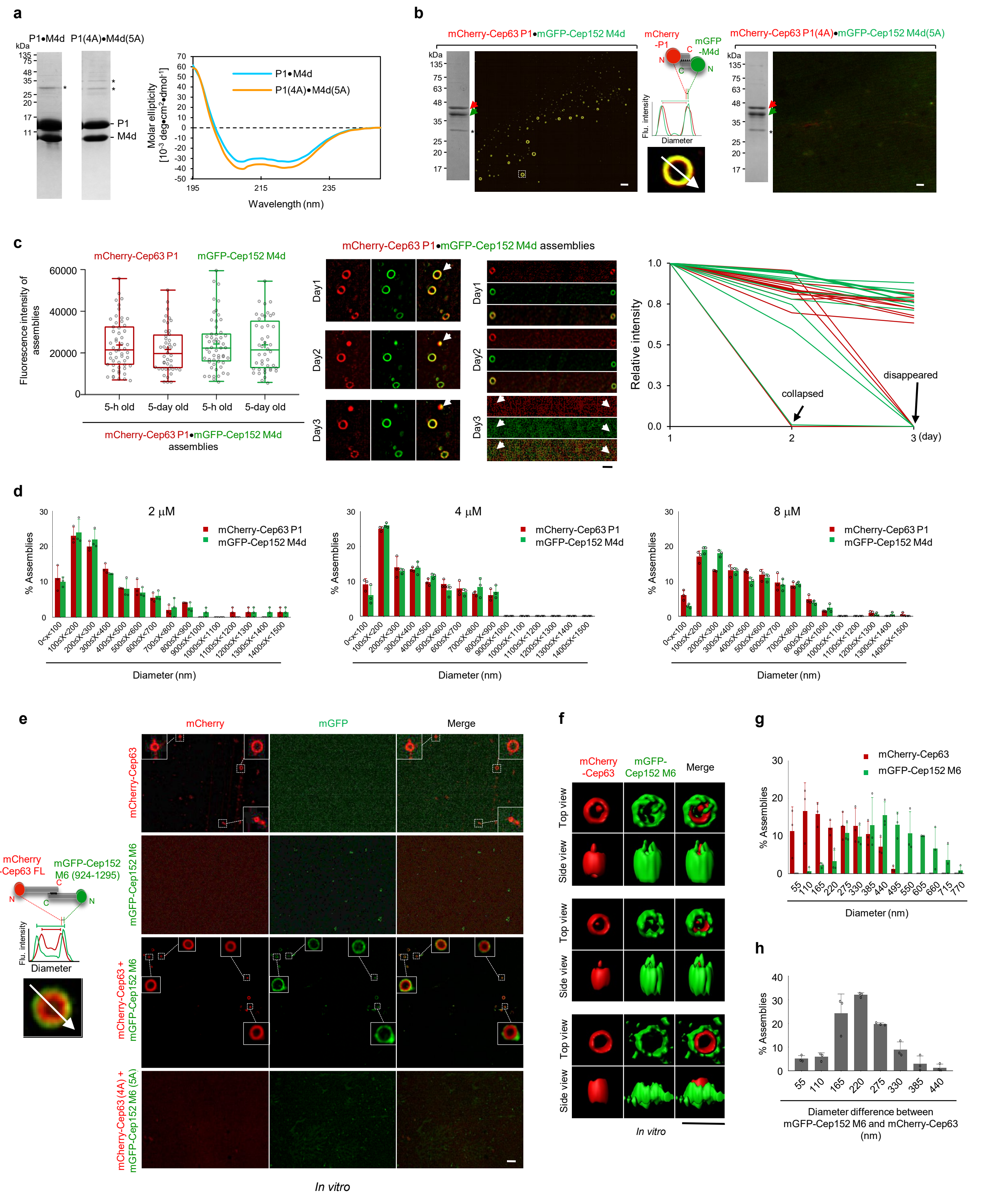

Supplementary Figure 2

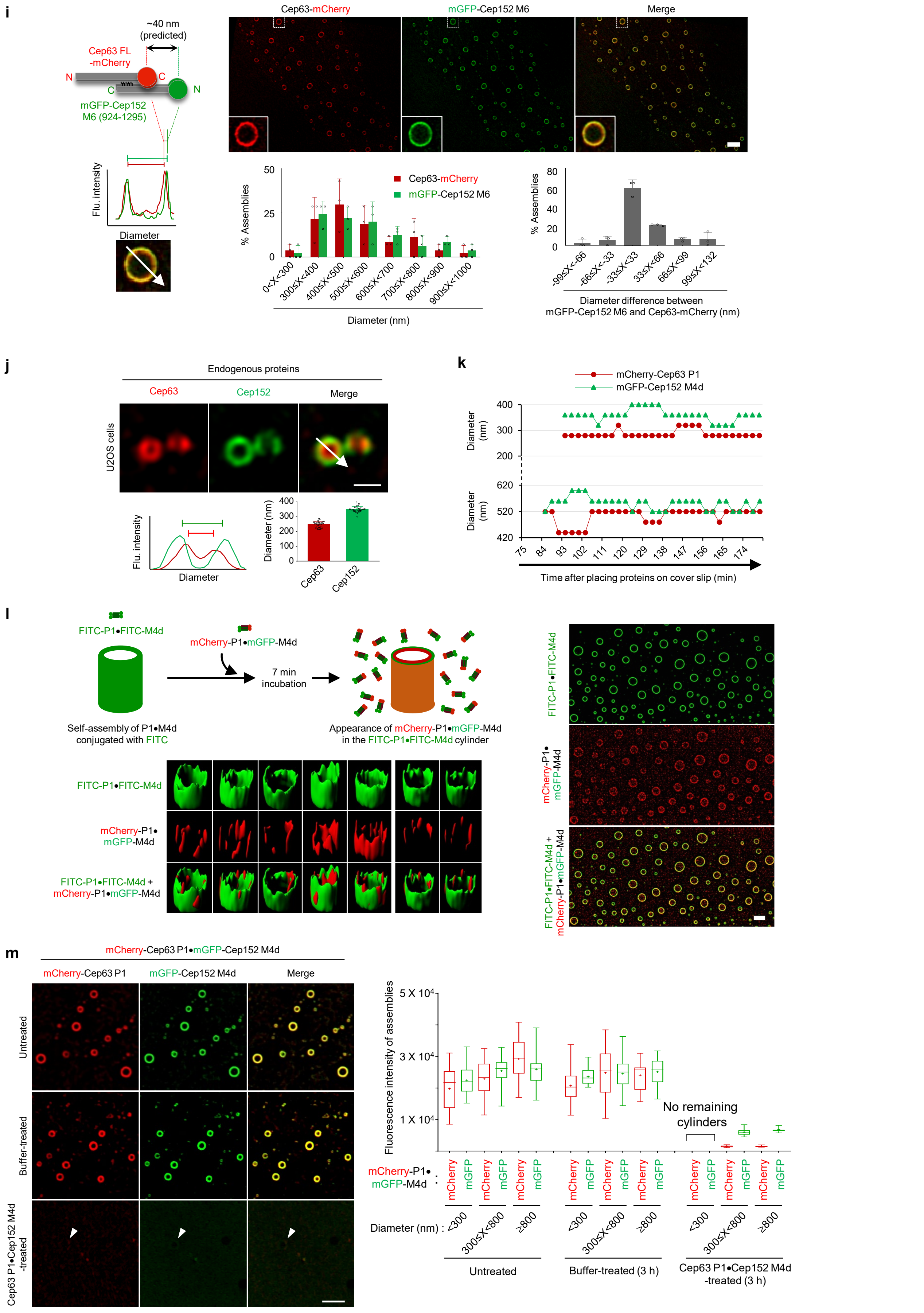

Supplementary Fig. 2. Cep63 and Cep152 self-assemble into a cylindrical architecture in a hydrophobic motif-dependent manner. a, Circular dichroism analysis of the Cep63 P1•Cep152 M4d complex and its corresponding P1(4A)•M4d(5A) hydrophobic mutant. Both spectra showed a strong broad negative band between 200 nm and 235 nm with the lowest points of the spectra at 208 nm and 222 nm. Asterisks on Coomassie-stained gels (left), contaminating proteins. **b,** 3D-SIM of the cylindrical self-assemblies generated by the mCherry-Cep63 P1•mGFP-Cep152 M4d complex, but not its corresponding hydrophobic mutant, *in vitro*. The schematic diagram (middle) of the mCherry-Cep63 P1(424–541)•mGFP-Cep152 M4d(1205–1295) complex showing a short inter-distance between mCherry and mGFP fluorescence is drawn based on the crystal structure of the heterotetrameric P1b5•M4e15 complex in Fig. 3b. The enlarged image with line-scan plots illustrates how the diameters of the mCherry and mGFP fluorescence were measured. Line-scan plots were generated in the arrowed direction. Asterisks on Coomassie-stained gels, degradation products. **c,** 3D-SIM analyses and signal quantifications for the mCherry-Cep63 P1•mGFP-Cep152 M4d self-assembly placed under the assembly buffer (see Methods) at RT for the indicated lengths of time. Fluorescence intensities for the images acquired under the same laser power and exposure time were quantified from a total of 68 assemblies obtained from three independent experiments. Some of the selected images (middle) and their signal intensities (right) are shown over time. Arrows, self-assemblies either collapsed in day 2 or disappeared in day 3. **d,** The dimension of mCherry-Cep63 P1•mGFP-Cep152 M4d self-assemblies generated under indicated concentrations were quantified as shown in Fig. 2d from a total of 216 (for 4 μM), 204 (for 8 μM), or 73 (for 2 μM) obtained from two independent experiments. **e,** 3D-SIM analysis for the *in vitro* self-assembled mCherry-Cep63•mGFP-Cep152 M6(924–1295) complex. mCherry-Cep63 FL, mGFP-Cep152 M6, and their corresponding hydrophobic mutants (4A and 5A, respectively) were individually purified, and their abilities to generate a self-assembly either alone or as a complex were examined. The schematic diagram (left) illustrates a greatly increased mCherry-mGFP distance in the longer mCherry-Cep63•mGFP-Cep152 M6(924–1295) complex (compare with the shorter mCherry-Cep63 P1(424–541)•mGFP-Cep152 M4d(1205–1295) complex). **f,** Three representative examples of surface-rendered self-assemblies generated by the mCherry-Cep63•mGFP-Cep152 M6(924–1295) complex in (**c**) are shown. Note that the cylinders made of this longer complex appeared to be less stable than the cylinders generated from the shorter complex in Fig. 2c. **g**, **h**, The diameter of mCherry-Cep63 and mGFP-Cep152 M6 fluorescence (**g**) and the differences in their diameters (**h**) were quantified similarly as in Fig. 2d, f from a total of 472 self-assemblies obtained from three independent experiments (169, 153, and 150 self-assemblies from each experiment). **i,** 3D-SIM analysis for the self-assemblies generated by Cep63 C-terminally tagged with mCherry (i.e., Cep63-mCherry) and mGFP-Cep152 M6(924–1295). The schematic diagram (left) illustrates a greatly shortened mCherry-mGFP distance (~40 nm if the marked region were made of α-helix as predicted by the PSIPRED server). A representative 3D-SIM image is shown (upper right). The differences in their diameters were quantified similarly as in Fig. 2d, f from a total of 56 self-assemblies obtained from two independent experiments (lower right). **j,** 3D-SIM analysis for asynchronously growing U2OS cells immunostained with a Cep63 antibody against the total protein and an N-terminal Cep152 antibody. A line-scan plot was generated in the direction of the arrow. The diameters of Cep63 and Cep152 were individually quantified from a total of 48 toroids. The diameter difference for endogenous Cep63 and Cep152 was estimated to be ~102 nm when using a Cep63 antibody generated against the FL protein (Note that the *in vitro* diameter difference for mCherry-Cep63 and mGFP-Cep152 was 165–275 nm; Supplementary Fig. 2h). Given that Cep63 is oriented in the C-terminal outward fashion, the fluorescent signal from the total anti-Cep63 antibody may increase the Cep63's toroidal diameter in comparison to that with the N-terminal mCherry-tagged Cep63, thus resulting in a decrease in the diameter difference between Cep63 and Cep152 fluorescent signals. **k,** Analyses of two additional SIM-TIRF time-lapse movies showing that, similar to the result shown in Fig. 2i, the diameters of the mCherry-Cep63 P1•mGFP-Cep152 M4d self-assemblies with two different sizes (~300 nm and ~520 nm) also remain unchanged over time. **l,** 3D-SIM analysis for FITC-decorated Cep63 P1•Cep152 M4d self-assemblies incubated with the mCherry-Cep63 P1•mGFP-Cep152 M4d complex provided into the assembly buffer. The self-assemblies generated with 6 μM of the Cep63 P1•Cep152 M4d complex for 16 h were reacted with FITC (see Methods for details). After washing out unassembled Cep63 P1•Cep152 M4d complexes and unreacted FITC, the self-assemblies were further incubated with 6 μM of the mCherry-Cep63 P1•mGFP-Cep152 M4d complex for 7 min (upper left). The resulting sample was washed and subjected to 3D-SIM (right). Bar, 1 μm. Representative examples of surface-rendered cylinders are shown (lower left). Note that the first five images show mCherry fluorescence preferentially detected at the bottom of the cylinders, while the last two images show the mCherry fluorescence mainly from the top of the cylinders. **m**, 3D-SIM analyses (left) and signal quantifications (right) for mCherry-Cep63 P1•mGFP-Cep152 M4d self-assemblies left untreated, buffer-treated, or treated with the nonfluorescent Cep63 P1●Cep152 M4d complex. Bar, 1 μm. Fluorescence intensities were quantified after classifying cylindrical assemblies into three groups according to their measurable sizes in diameter. Total numbers of cylinders counted are: n = 94 (untreated), n = 91 (buffer-treated), and n = 29 (the Cep63 P1●Cep152 M4d complex-treated). Arrowhead, a remnant of fluorescent signals rarely detected under the Cep63 P1●Cep152 M4d complex-treated condition. Center line, median; cross, mean; box limits, upper and lower quartiles; whiskers, maximum/minimum of the data.

Supplementary Figure 3

a

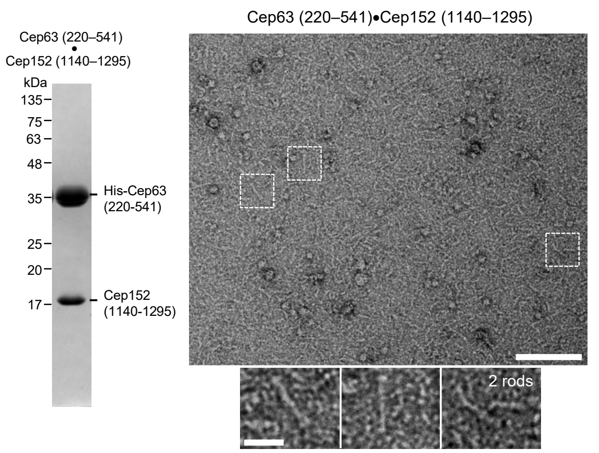

b

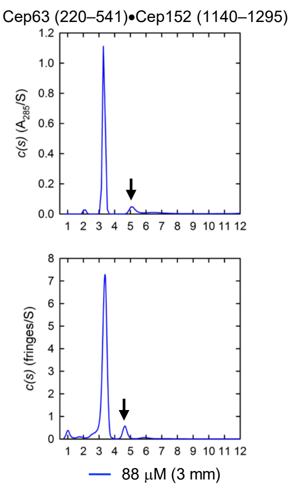

c

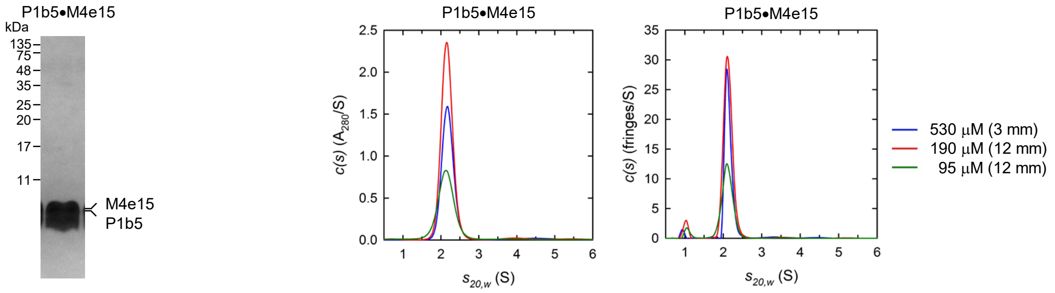

d

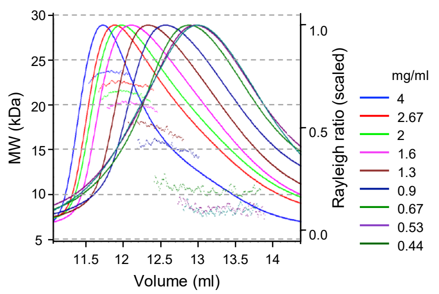

Molar concentration (µM)	MW (kDa)
314.96	23.40
209.97	22.30
157.48	21.20
125.98	20.00
104.99	17.40
69.99	15.40
52.49	10.60
41.99	8.50
35.00	8.20

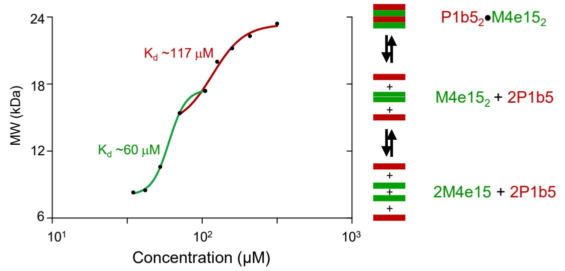

e

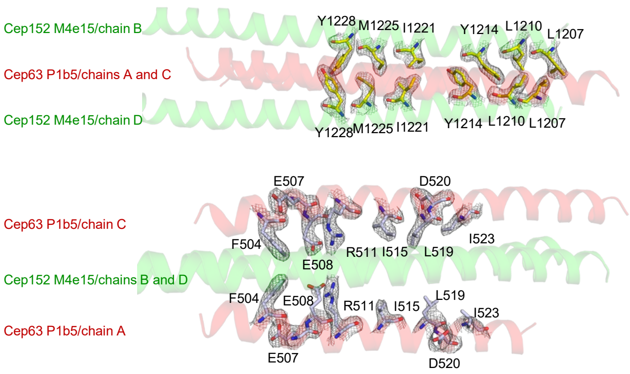

f

Fig. 3d, top

Chain ID	Residue	Chain ID	Residue	Interaction
B	R1253	D	R1253	Hydrogen Bond
B	D1250	D	D1250	Hydrogen Bond
B	E1243	D	R1246	Salt Bridge
B	S1232	D	Y1228	Hydrogen Bond
B	Y1228	D	Y1228	Hydrophobic
B	M1225	D	M1225	Hydrophobic
B	I1221	D	I1221	Hydrophobic
B	Y1214	D	Y1214	Hydrophobic
B	L1210	D	L1210	Hydrophobic
B	L1207	D	L1207	Hydrophobic

Fig. 3d, middle

Chain ID	Residue	Chain ID	Residue	Interaction
A	F504	B	A1236	Hydrophobic
A	L505	D	A1236	Hydrophobic
A	E507	B	R1235	Salt Bridge
A	E508	B	R1235	Salt Bridge
A	E509	D	K1233	Salt Bridge
A	R511	B	Y1228	Hydrogen Bond
A	R511	B	S1232	Hydrogen Bond
A	S512	D	I1229	Hydrophobic
A	I515	B	M1225	Hydrophobic
A	L516	D	L1226	Hydrophobic
A	L516	D	I1229	Hydrophobic
A	L519	B	I1221	Hydrophobic
A	D520	D	K1222	Salt Bridge
A	H522	B	D1224	Salt Bridge
A	I523	D	V1218	Hydrophobic
A	L526	D	Y1214	Hydrophobic
A	S530	B	Y1214	Hydrogen Bond

Fig. 3d, bottom

Chain ID	Residue	Chain ID	Residue	Interaction
C	T502	B	V1240	Hydrophobic
C	F504	B	A1236	Hydrophobic
C	L505	B	A1236	Hydrophobic
C	E507	D	R1235	Salt Bridge
C	E508	D	R1235	Salt Bridge
C	E509	B	K1233	Salt Bridge
C	R511	B	S1232	Hydrogen Bond
C	S512	D	I1229	Hydrophobic
C	I515	B	M1225	Hydrophobic
C	L516	D	L1226	Hydrophobic
C	L519	B	I1221	Hydrophobic
C	D520	D	K1222	Salt Bridge
C	H522	B	D1224	Salt Bridge
C	I523	B	V1218	Hydrophobic
C	E525	D	K1220	Salt Bridge
C	L526	B	V1218	Hydrophobic
C	E529	D	K1216	Salt Bridge
C	S530	B	Y1214	Hydrogen Bond
C	T533	D	E1209	Hydrogen Bond
C	V534	B	E1208	Hydrogen Bond

g

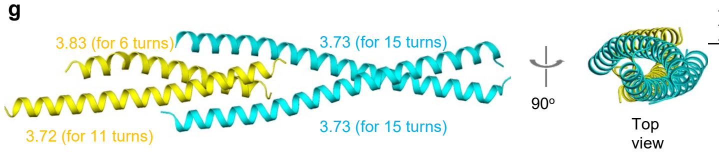

The *Drosophila* Cnn LZ-CM2 complex

Supplementary Figure 3

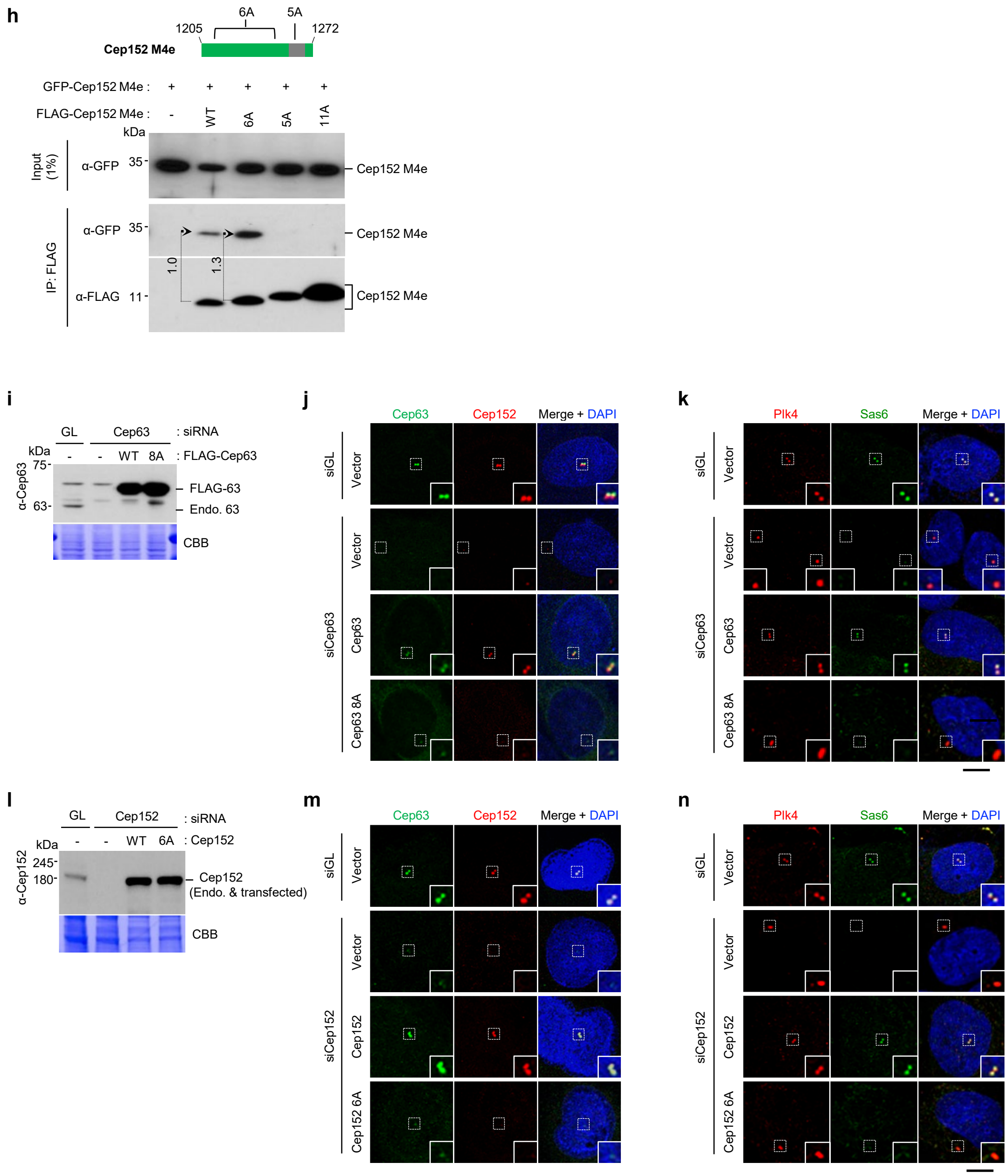

Supplementary Fig. 3. The Cep63•Cep152 complex and the biological function of the heterotetrameric Cep63–Cep152 interaction. a, The Cep63(220–541)•Cep152(1140–1295) complex stained with Coomassie and an example of uncropped transmission electron micrograph showing after negative staining. Dotted boxes, areas of enlargement. **b**, Sedimentation velocity analysis of the Cep63(220–541)•Cep152(1140–1295) complex at 88 μM showing a dominant species at 3.30 S that has an estimated MW of 110 kDa, suggestive of a 2:2 heterotetrameric complex (expected MW of 112.54 kDa). Both c(*s*) plots exhibit the presence of a component at 4.7 – 5.2 S (~8% of the signal; arrow). Based on scaling laws, one would expect a dimer to sediment approximately 1.5 times as fast as the monomer [4]. Therefore, the species at ~5 S may likely represent a dimer of the 3.30 S heterotetramer (i.e., heterooctamer). **c**, Sedimentation velocity analyses of the Cep63 P1b5•Cep152 M4e15 complex. The complex stained with Coomassie is shown (left). Data show the presence of a single species at 2.14 S with an estimated MW of 25 kDa suggestive of a 2:2 heterotetrameric complex (expected MW 25.56 kDa). **d,** Overlay of SEC-MALS chromatograms of the Cep63 P1b5•Cep152 M4e15 complex at various

concentrations (left) and plot of average MW from SEC-MALS analysis as a function of protein concentration (right). The apparent biphasic mode of MW changes was interpreted based on the crystal structure model (see Fig. 3c) in such a way that a stronger interaction (K_d ~60 μM) was accredited to the core M4e15-M4e15 interaction of the four-helix bundle, while a weaker interaction (K_d ~117 μM) was assigned to two P1b5 chains that bind to the M4e15 dimer. **e,** Crystal structure of the Cep63 P1b5•Cep152 M4e15 complex showing electron density maps for six Cep152 (top) and eight Cep63 (bottom) residues critical for heterotetramer formation. The 2|Fo|-|Fc| electron density is contoured to 1.0 σ (black mesh). The carbon (yellow or grey), oxygen (red), and nitrogen (blue) atoms for these residues are colored. Two chains of P1b5 (red) and two chains of M4e15 (green) are shown in the background. **f,** Important Cep152 M4e15-Cep152 M4e15 and Cep152 M4e15-Cep63 P1b5 interactions shown in Fig. 3d are summarized. **g,** Cnn, which shows an average α-helical periodicity of 3.75 residues per helical turn, exhibits an unusual left-handed supercoil. This is in sharp contrast to other coiled-coils with periodicities of 3.75 residues, which are typically right-handed [5]. The axes of the four α-helices of the Cep63 P1b5•Cep152 M4e15 complex (an average α-helical periodicity of 3.70 residues) are straight, rather than wounding one another in a supercoil arrangement (Fig. 3c). **h,** IP and immunoblotting using transfected HEK293 cells. The diagram shows the region where marked mutations are introduced. Gray box, the hydrophobic motif present in Cep152 M4e; Numbers, relative coimmunoprecipitation efficiencies. The 6A mutations, which disrupt the heterotetrameric complex formation in the presence of Cep63 in Fig. 3g, do not impair the homodimeric M4e–M4e interaction. Rather, the 5A mutations in the Cep152 hydrophobic motif disrupt the M4e–M4e interaction. These data strongly suggest that while the hydrophobic motif is normally involved in the dimeric M4e–M4e interaction, the M4e15 region (the area marked with 6A) functions in concert with the P1b5 region of Cep63 to cooperatively generate a heterotetrameric α-helical bundle (Fig. 3g). **i–n,** Analysis of U2OS cells stably expressing the indicated constructs after silencing for control luciferase (siGL), Cep63 (siCep63) or Cep152 (siCep152). The resulting cells were immunoblotted to examine the level of exogenous Cep63 or Cep152 expression (**i** and **l**) and immunostained for confocal microscopy (**j, k, m, n**). Images were analysed to generate the data shown in Fig. 3h–k. CBB, Coomassie-stained membrane; dotted boxes, areas of enlargement. The sub-centrosomal localization of Plk4 changes from a ring state to a dot state depending on Plk4's activation state [6,7]. Furthermore, the level of recruited Plk4 rather increases in Cep152-depleted cells because of the presence of another Plk4-recruiting scaffold, Cep192 [6,8]. For these reasons, we did not quantify the level of Plk4 recruitment in these samples.

Supplementary Figure 4

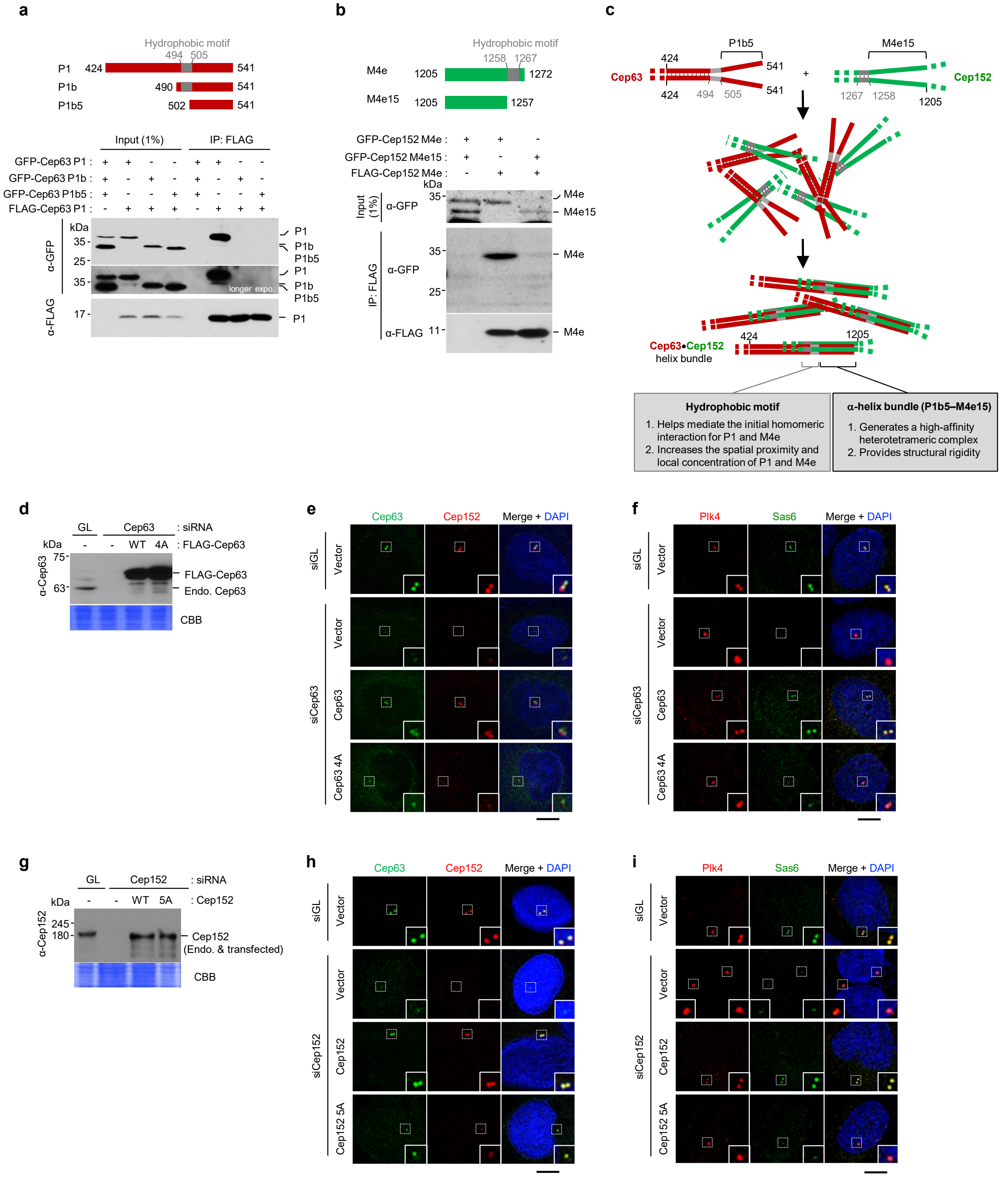

Supplementary Fig. 4. The role of hydrophobic motifs in the Cep63–Cep152 interaction and its significance in centriole duplication. a, b, IP and immunoblotting using transfected HEK293 cells. The schematic diagram shows various Cep63 and Cep152 fragments analyzed for Cep63–Cep63 (**a**) and Cep152–Cep152 (**b**) interactions. **c,** Model illustrating the mechanism of how Cep63 and Cep152 interact to form a heterotetrameric α-helical bundle. The results in **(a)** and **(b)** suggest that while the Cep152 M4d hydrophobic motif plays an important role in promoting the homomeric Cep152–Cep152 interaction, the Cep63 P1 hydrophobic motif does not appear to directly mediate its homomeric interaction. Rather, it contributes significantly to the Cep63 P1b-Cep152 M4e interaction as demonstrated in Supplementary Fig. 1e, suggesting that the Cep63 P1 hydrophobic motif promotes the heteromeric Cep63–Cep152 interaction. Thus, both homomeric and heteromeric interactions observed with the hydrophobic motifs in Cep63 and Cep152 may help increase their local concentrations through clustering. The four-helix bundle generated through tightly engaged M4e15–M4e15 and P1b5•M4e15 interactions (see Fig. 3d) may provide structural rigidity to the bundle. Based on the data in (**a**) and (**b**), the hydrophobic motif in Cep152 is schematically drawn to mediate the dimeric Cep152–Cep152 interaction, whereas the hydrophobic motif in Cep63 is not. **d–i,** Analysis of U2OS cells stably expressing the indicated constructs after silencing for control luciferase (siGL), Cep63 (siCep63), or Cep152 (siCep152). The resulting cells were immunoblotted to examine the level of exogenous Cep63 or Cep152 expression (**d** and **g**) and immunostained for confocal microscopy (**e, f, h, i**). Images were analysed to generate Fig. 4c–f. CBB, Coomassie-stained membrane; dotted boxes, areas of enlargement.

Supplementary Figure 5

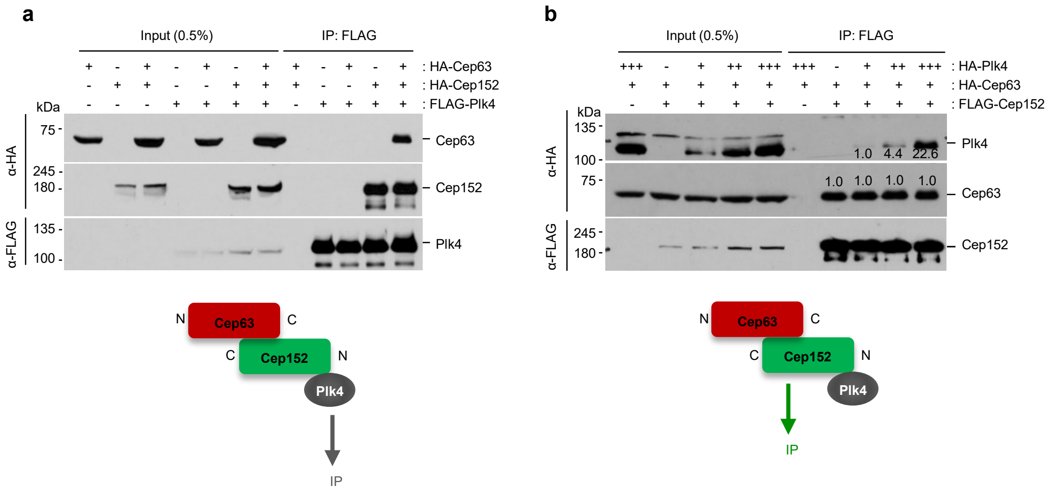

Supplementary Fig. 5. Cep63 associates with Plk4 via Cep152. a, b, IP and immunoblotting using transfected HEK293 cells. The schematic diagram illustrates that Cep152 bridges the association between Cep63 and Plk4. Numbers, relative signal intensities.

Supplementary Figure 6

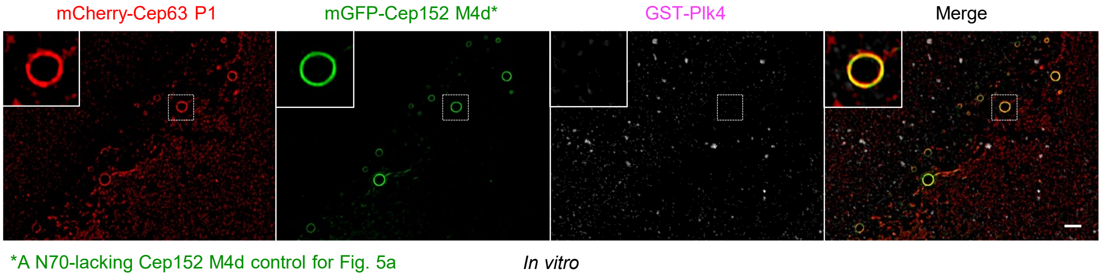

Supplementary Fig. 6. The N70 motif of Cep152 is required for the recruitment of Plk4 to Cep63•Cep152 self-assemblies. 3D-SIM analysis showing that Cep63 P1•Cep152 M4d self-assemblies lacking the N70 (a minimal Plk4-binding motif) of Cep152 fail to recruit Plk4 *in vitro*. This is in contrast to the Cep63 P1•Cep152 N70-M4d self-assemblies shown in Fig. 5a. This control experiment was carried out at the same time as the results shown in Fig. 5a.

Supplementary Figure 7

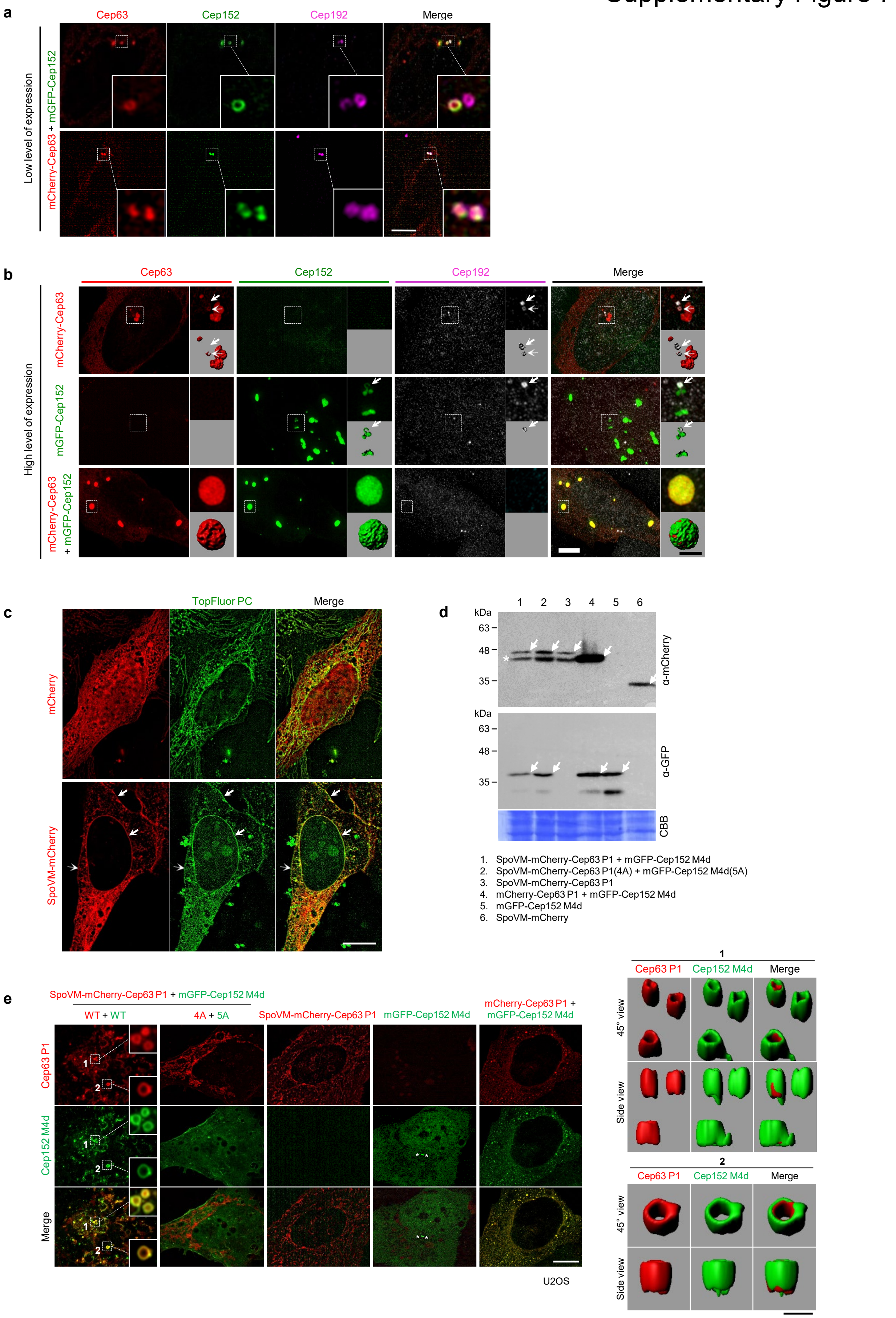

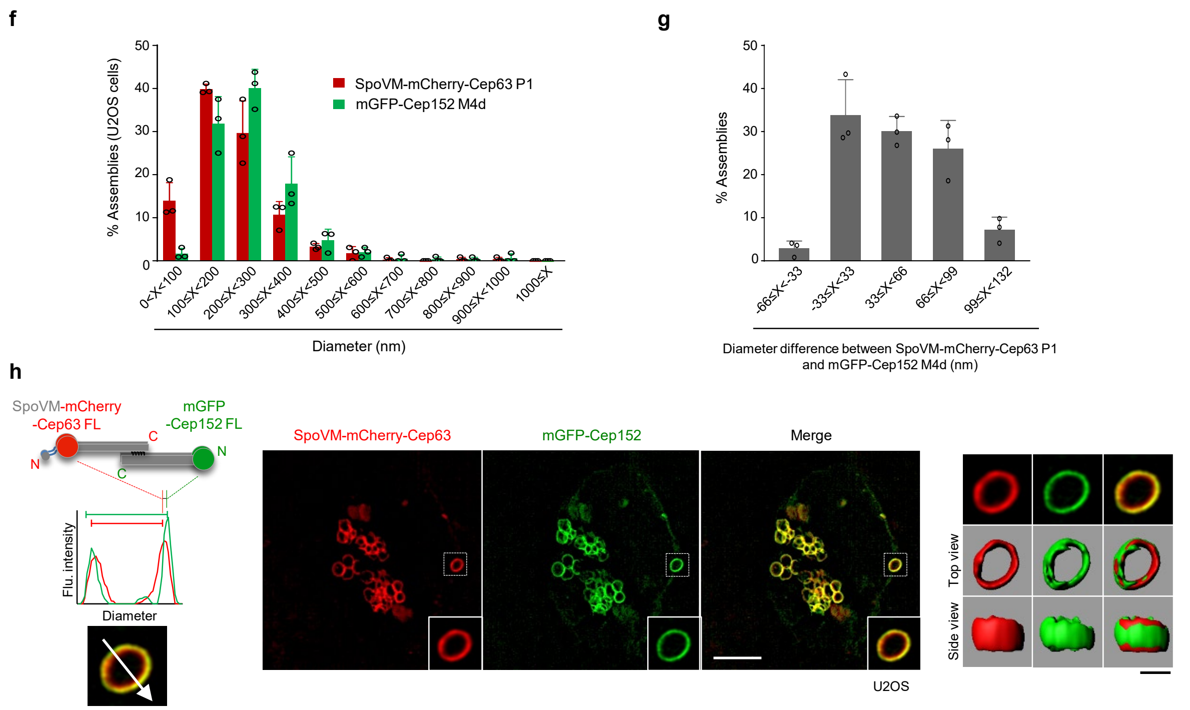

Supplementary Fig. 7. *In vivo* formation of a cylindrical Cep63•Cep152 self-assembly using a membrane-targeted Cep63. a, b, 3D-SIM analysis for immunostained U2OS cells cotransfected with mCherry-Cep63 and mGFP-Cep152. The location of endogenous centrioles is marked by anti-Cep192 signals. Cells expressing low (**a**) or high (**b**) levels of mCherry-Cep63 and mGFP-Cep152 proteins are shown. When expressed at a high level (**b**), these proteins cooperate to generate a spherical assembly in cytosol (third panel). Surface rendering in (**b**) shows that cytosolic mCherry-Cep63 and mGFP-Cep152 coacerbate to generate architecturally uncharacterizable aggregates. Dotted boxes, areas of enlargement; arrows in (**b**), endogenous centrosomes. **c,** 3D-SIM analysis for transfected U2OS cells after staining with TopFluor phosphatidylcholine (PC). Arrows indicate membrane-targeted SpoVM-mCherry signals colocalized with TopFluor PC. **d,** Immunoblotting analysis for U2OS cells transfected with the indicated constructs. Arrows, signals corresponding to the expected sizes of the constructs expressed; CBB, Asterisk, degradation product. Coomassie-stained membrane. **e,** *In vivo* formation of a higher-order cylindrical architecture by membrane-targeted SpoVM-mCherry-Cep63 P1 and mGFP-Cep152 M4d in U2OS cells. 3D-SIM analysis was carried out and the assemblies indicated by the dotted boxes were subjected to surface rendering (right). **f, g,** The diameter of mCherry-Cep63 P1 and mGFP-Cep152 M4d fluorescence (**f**) and the differences in their diameters (**g**) for assemblies in U2OS cells were quantified from three independent experiments (128, 112, and 97 assemblies from each experiment), as described in Fig. 2d, f. Bars, s.d. **h,** 3D-SIM showing an *in vivo* cylindrical architecture generated by membrane-targeted SpoVM-mCherry-Cep63 FL and mGFP-Cep152 FL in U2OS cells. The schematic diagram shows the SpoVM-mCherry-Cep63•mGFP-Cep152 complex. Line-scan plot (left) was generated in the arrowed direction. The dotted box indicates the assembly subjected to surface rendering (right).

Supplementary Figure 8

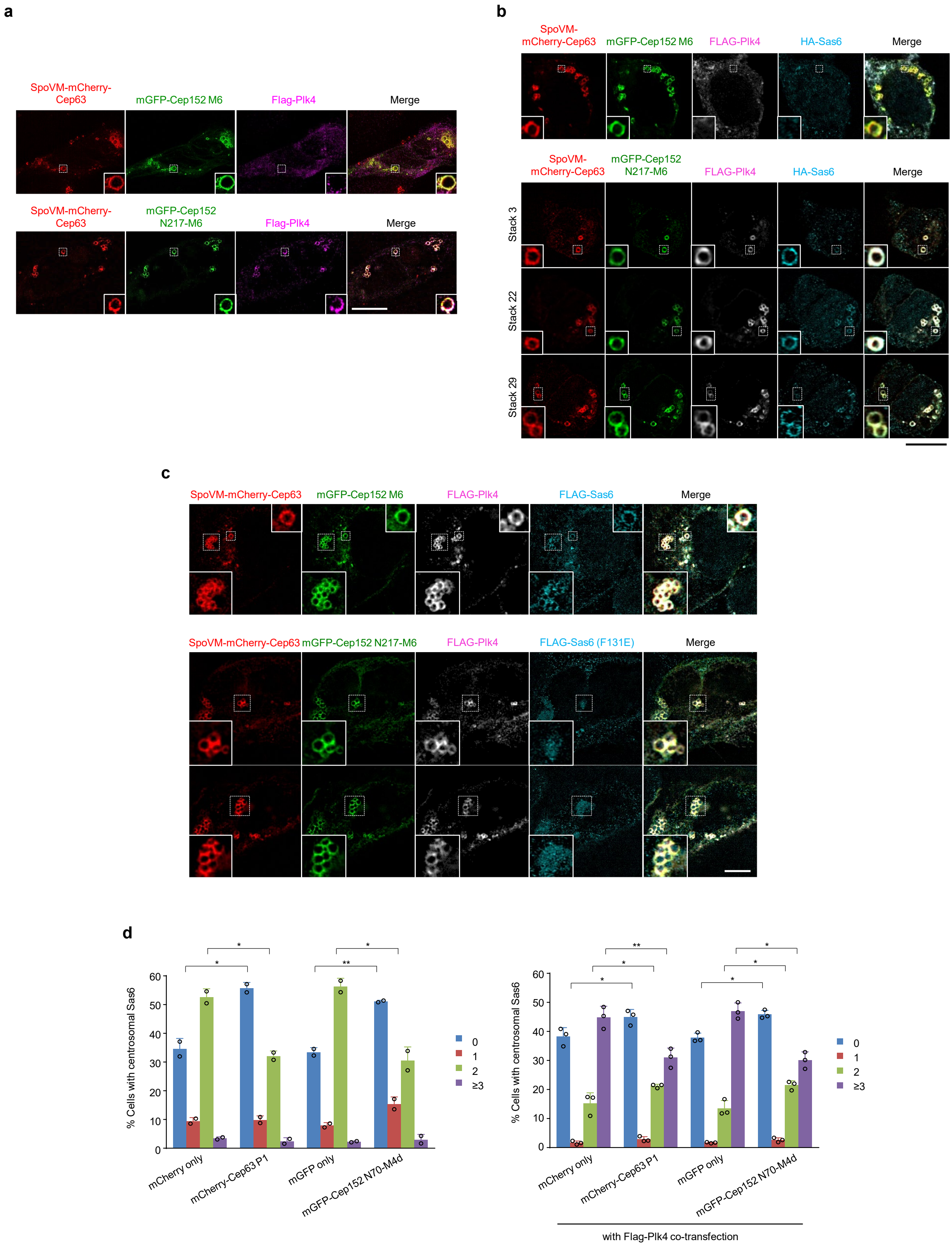

Supplementary Fig. 8. ***In vivo*** **recruitment of the downstream components of the Cep63•Cep152 self-assembly. a, b,** 3D-SIM analysis for immunostained U2OS cells cotransfected with the indicated constructs. To examine the ability of Cep63•Cep152 self-assemblies to recruit FLAG-Plk4 and its downstream component Sas6, either a Plk4 binding-deficient mGFP-Cep152 M6 or a Plk4-binding N217 [6]-containing mGFP-Cep152 N217-M6 was used (see details in Methods). Note that N217-containing assemblies efficiently recruited not only Plk4 (**a**) but also Sas6 (**b**). Dotted boxes, areas of enlargement. **c,** 3D-SIM conducted essentially as in **b** except that FLAG-Sas6 or its respective oligomerization-defective F131E mutant was used to replace the HA-Sas6 construct in **b**. To detect FLAG-Plk4 and FLAG-Sas6 signals, anti-Plk4 and anti-Sas6 antibodies, respectively, were used with appropriate secondary antibodies. Note that the Sas6 (F131E) mutant does not appear to be efficiently recruited to the region where ring-like Plk4 signals were found, nor does it exhibit focused Sas6 signals. **d,** U2OS cells transfected with the indicated constructs were immunostained with anti-Sas6 and anti-Plk4 antibodies. The number of centrosomal Sas6 colocalized with Plk4 signals was quantified from either two (left) or three (right) independent experiments (59 to 129 cells/sample/experiment). Error bars, standard deviation; *, $P < 0.05$; **, $P < 0.01$ (unpaired two-tailed t-test). Note that under both conditions with or without Plk4 coexpression, either Cep63 P1 or Cep152 N70-M4d alone significantly diminished the fraction of cells with centrosome-localized Sas6 dot signals, suggesting that it blocks centriole duplication in a dominant-negative manner.

Supplementary Figure 9

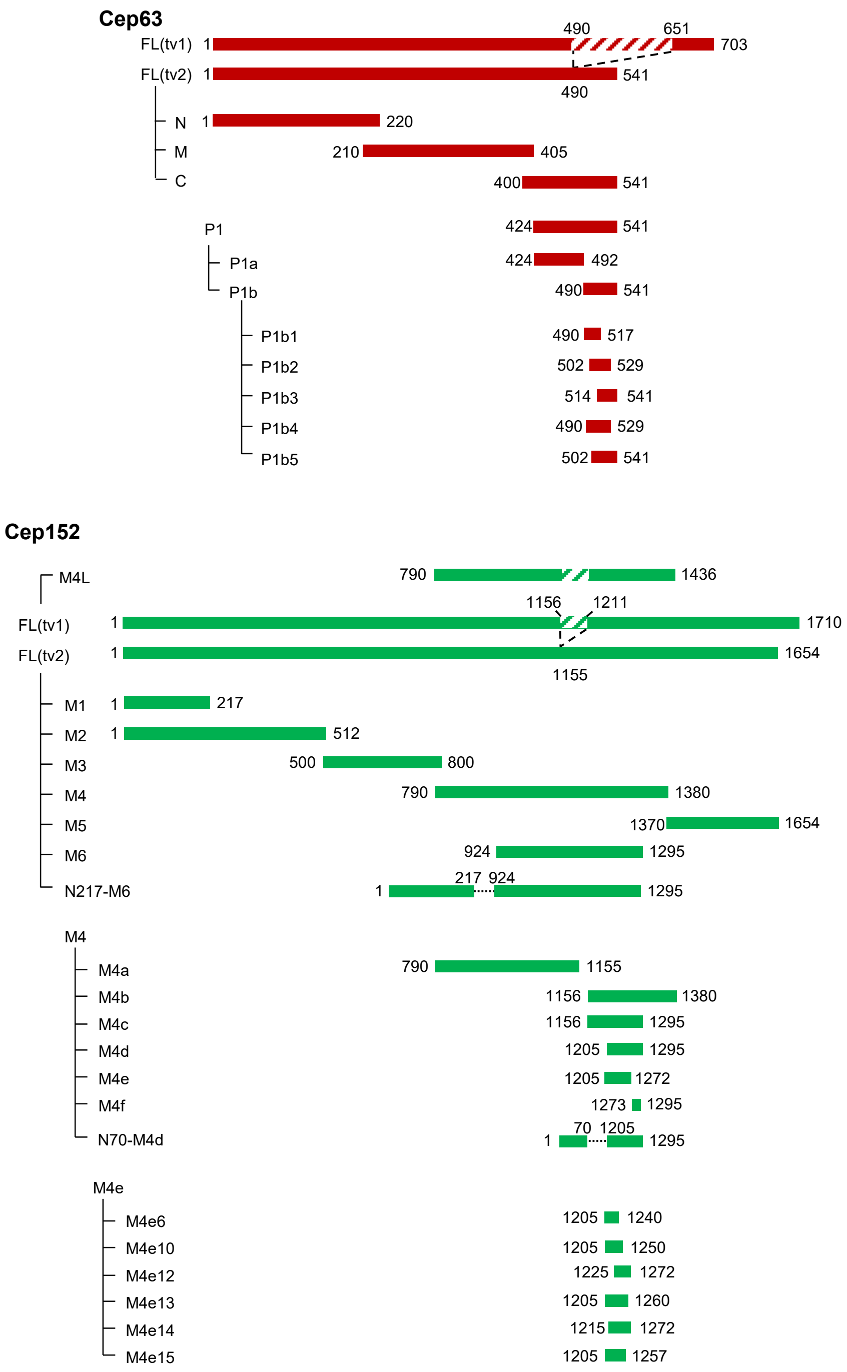

Supplementary Fig. 9. Summary of all the Cep63 and Cep152 constructs used in this study.

Supplementary references

1 Kyte, J. & Doolittle, R. F. A simple method for displaying the hydropathic character of a protein. *J. Mol. Biol.* **157**, 105-132 (1982).

2 Lawo, S., Hasegan, M., Gupta, G. D. & Pelletier, L. Subdiffraction imaging of centrosomes reveals higher-order organizational features of pericentriolar material. *Nat. Cell Biol.* **14**, 1148-1158. (2012).

3 Park, S. Y. *et al.* Molecular basis for unidirectional scaffold switching of human Plk4 in centriole biogenesis. *Nat. Struct. Mol. Biol.* **21**, 696-703, doi:10.1038/nsmb.2846 (2014).

4 Brown, P. H. & Schuck, P. Macromolecular size-and-shape distributions by sedimentation velocity analytical ultracentrifugation. *Biophys. J.* **90**, 4651-4661, doi:10.1529/biophysj.106.081372 (2006).

5 Lupas, A. N. & Bassler, J. Coiled Coils - A Model System for the 21st Century. *Trends Biochem. Sci.* **42**, 130-140, doi:10.1016/j.tibs.2016.10.007 (2017).

6 Kim, T.-S. *et al.* Hierarchical recruitment of Plk4 and regulation of centriole biogenesis by two centrosomal scaffolds, Cep192 and Cep152. *Proc. Natl. Acad. Sci. USA* **110**, E4849-4857 (2013).

7 Ohta, M. *et al.* Direct interaction of Plk4 with STIL ensures formation of a single procentriole per parental centriole. *Nat. Commun.* **5**, 5267, doi:10.1038/ncomms6267 (2014).

8 Sonnen, K. F., Gabryjonczyk, A. M., Anselm, E., Stierhof, Y. D. & Nigg, E. A. Human Cep192 and Cep152 cooperate in Plk4 recruitment and centriole duplication. *J. Cell Sci.* **126**, 3223-3233. (2013).

9 Elbashir, S. M. *et al.* Duplexes of 21-nucleotide RNAs mediate RNA interference in cultured mammalian cells. *Nature* **411**, 494-498. (2001).

10 Zhao, H. *et al.* The Cep63 paralogue Deup1 enables massive de novo centriole biogenesis for vertebrate multiciliogenesis. *Nat. Cell Biol.* **15**, 1434-1444. (2013).

11 Cizmecioglu, O. *et al.* Cep152 acts as a scaffold for recruitment of Plk4 and CPAP to the centrosome. *J. Cell Biol.* **191**, 731-739. (2010).

Supplementary Table 1. Data collection, phasing and refinement statistics for SAD (SeMet) structures

	Cep63 P1b5(502–541)-GGGSE -Cep152M4e10(1205-1250) (Native)	Cep63 P1b5(502–541)-GGGSE -Cep152M4e10(1205-1250) (SeMet derivative)
**Data collection***		
Space group	$P2_12_12_1$	P4222
Cell dimensions		
a, *b*, *c* (Å)	44.40, 44.42, 225.9	44.44, 44.44, 226.13
α, β, γ (°)	90.0, 90.0, 90.0	90.0, 90.0, 90.0
Wavelength	1.0000	0.9793
Resolution (Å)	19.93 - 2.50 (2.54 - 2.50)**	50 - 2.30 (2.34 - 2.30)
R_{sym} or R_{merge}	0.064 (0.299)	0.055 (0.693)
$I / \sigma I$	15.82 (1.82)	25.4 (1.1)
Completeness (%)	93.17 (72.72)	96.10 (76.00)
Redundancy	4.9 (2.9)	5.7 (2.3)
**Refinement**		
Resolution (Å)	19.93 - 2.50	
No. reflections	15588	
R_{work} / R_{free}	0.248/0.284	
No. atoms		
Protein	3007	
Ligand/ion	0	
Water	37	
B-factors	58.37	
Protein	58.40	
Ligand/ion	0	
Water	58.20	
R.m.s deviations		
Bond lengths (Å)	0.009	
Bond angles (°)	1.26	

*All data sets collected from single crystals

**Values in parentheses are for highest-resolution shell.

Supplementary Table 2. Plasmid constructs used in this study

List	Protein	Residues	Expression vector	Restriction site
pKM4858	Cep63	1–541	pCI-neo-GFP vector (pKM3828)	PmeI, NotI
pKM4861	Cep63 P1	424–541	pCI-neo-GFP vector (pKM3828)	PmeI, NotI
pKM4859	Cep63 P1a	424–492	pCI-neo-GFP vector (pKM3828)	PmeI, NotI
pKM4860	Cep63 P1b	490–541	pCI-neo-GFP vector (pKM3828)	PmeI, NotI
pKM5118	Cep63 P1b1	490–517	pCI-neo-GFP vector (pKM3828)	PmeI, NotI
pKM5119	Cep63 P1b2	502–529	pCI-neo-GFP vector (pKM3828)	PmeI, NotI
pKM5120	Cep63 P1b3	514–541	pCI-neo-GFP vector (pKM3828)	PmeI, NotI
pKM5186	Cep63 P1b4	490–529	pCI-neo-GFP vector (pKM3828)	PmeI, NotI
pKM5154	Cep63 P1b5	502–541	pCI-neo-GFP vector (pKM3828)	PmeI, NotI
pKM3841	Cep152	1–1654	pEGFP-C1 vector	XhoI, SmaI
pKM4518	Cep152 M3	500–800	pEGFP-C1 vector	XhoI, SmaI
pKM4519	Cep152 M4	790–1380	pEGFP-C1 vector	XhoI, SmaI
pKM4520	Cep152 M5	1370–1654	pEGFP-C1 vector	XhoI, SmaI
pKM4844	Cep152 M4a	790–1155	pEGFP-C1 vector	XhoI, SmaI
pKM4845	Cep152 M4b	1156–1380	pEGFP-C1 vector	XhoI, SmaI
pKM4880	Cep152 M4c	1156–1295	pEGFP-C1 vector	XhoI, SmaI
pKM4881	Cep152 M4d	1205–1295	pEGFP-C1 vector	XhoI, SmaI
pKM4962	Cep152 M4e	1205–1272	pEGFP-C1 vector	XhoI, SmaI
pKM4963	Cep152 M4f	1273–1295	pEGFP-C1 vector	XhoI, SmaI
pKM5112	Cep152 M4e6	1205–1240	pEGFP-C1 vector	XhoI, SmaI
pKM5135	Cep152 M4e10	1205–1250	pEGFP-C1 vector	XhoI, SmaI
pKM5137	Cep152 M4e12	1225–1272	pEGFP-C1 vector	XhoI, SmaI
pKM5138	Cep152 M4e13	1205–1260	pEGFP-C1 vector	XhoI, SmaI
pKM5139	Cep152 M4e14	1215–1272	pEGFP-C1 vector	XhoI, SmaI
pKM4837	Cep152 M4L	790–1436	pEGFP-C1 vector	XhoI, SmaI
pKM4882	Cep152 M4La	790–1199	pEGFP-C1 vector	XhoI, SmaI
pKM4883	Cep152 M4Lb	1156–1436	pEGFP-C1 vector	XhoI, SmaI
pKM3561	Cep152 M1	1–217	pEGFP-C1 vector	SalI, SmaI
pKM3562	Cep152 M2	1–512	pEGFP-C1 vector	SalI, SmaI
pKM5012	Cep152ΔM4e	Δ1205–1272	pEGFP-C1 vector	EcoRI, SalI
pKM4952	Cep152ΔM4d	Δ1205–1295	pEGFP-C1 vector	EcoRI, SalI
pKM5471	Cep152 5A (L1260A, I1261A, L1263A, I1266A, L1267A)	1–1654	pKM3841	SalI, EcoRI
pKM2798	Cep63	1–541	pCI-neo-FLAG$_3$ vector (pKM2795)	PmeI, NotI
pKM5111	Cep63 P1	424–541	pCI-neo-FLAG$_3$ vector (pKM2795)	PmeI, NotI
pKM5010	Cep63 P1b	490–541	pCI-neo-FLAG$_3$ vector (pKM2795)	PmeI, NotI
pKM5779	Cep63 N	1–220	pCI-neo-FLAG$_3$ vector (pKM2795)	PmeI, NotI
pKM5780	Cep63 M	210–405	pCI-neo-FLAG$_3$ vector (pKM2795)	PmeI, NotI
pKM5781	Cep63 C	400–541	pCI-neo-FLAG$_3$ vector (pKM2795)	PmeI, NotI
pKM4988	Cep63ΔP1b	1–489	pCI-neo-FLAG$_3$ vector (pKM2795)	PmeI, NotI
pKM1235	Cep63	1–541	pCI-neo-HA vector (pKM1209)	EcoRV, NotI/ SmaI, NotI
pKM5650	Cep63 4A (L497A, I500A, F504A, L505A)	1–541	pKM2798	EcoRV, NotI
pKM5935	Cep63 8A (F504A, E507A, E508A, R511A, I515A, L519A, D520A, I523A)	1–541	pKM2798	EcoRV, NotI
pKM2809	Cep152	1–1654	pCI-neo-FLAG$_3$ vector	XhoI, SmaI

			(pKM2795)	
pKM4987	Cep152 M4e	1205–1272	pCI-neo-FLAG$_3$ vector (pKM2795)	PmeI, NotI
pKM5346	Cep152ΔM4e	Δ1205–1272	pCI-neo-FLAG$_3$ vector (pKM2795)	PmeI, NotI
pKM6080	Cep152 M4	790–1380	pCI-neo-FLAG$_3$ vector (pKM2795)	PmeI, XhoI
pKM6081	Cep152 M4 6A (L1207A, L1210A, Y1214A, I1221A, M1225A, Y1228A)	790–1380	pCI-neo-FLAG$_3$ vector (pKM2795)	PmeI, XhoI
pKM4235	Cep152	1–1654	pCI-neo-HA vector (pKM1209)	PmeI, NotI
pKM5966	Cep152 M4e	1205–1272	pCI-neo-HA vector (pKM1209)	PmeI, NotI
pKM6078	Cep152 M4	790–1380	pCI-neo-HA vector (pKM1209)	PmeI, XhoI
pKM6079	Cep152 M4 6A	790–1380	pCI-neo-HA vector (pKM1209)	PmeI, XhoI
pKM3855	Plk4	1–970	pCI-neo-HA vector (pKM1209)	PmeI, NotI
pKM4483	STIL	1–1287	pCI-neo-HA vector (pKM1209)	PmeI, NotI
pKM4381	SAS6	1-490	pcDNA3.1-HA vector	Cla I, Xba I
pKM5930	Cep152 6A	1–1654	pKM2809	SalI, NheI
pKM6076	Cep152 6A	1–1654	pKM4235	SalI, NheI
pKM6364	Cep152 M4e 6A (L1207A, L1210A, Y1214A, I1221A, M1225A, Y1228A)	1205–1272	pCI-neo-FLAG$_3$ vector (pKM2795)	PmeI, NotI
pKM6365	Cep152 M4e 5A (L1260A, I1261A, L1263A, I1266A, L1267A)	1205–1272	pCI-neo-FLAG$_3$ vector (pKM2795)	PmeI, NotI
pKM6363	Cep152 M4e 11A (L1207A, L1210A, Y1214A, I1221A, M1225A, Y1228A, L1260A, I1261A, L1263A, I1266A, L1267A)	1205–1272	pCI-neo-FLAG$_3$ vector (pKM2795)	PmeI, NotI
pKM3445	Plk4		pCI-neo-FLAG$_3$ vector (pKM2795)	PmeI, NotI
pKM4961	mCherry	1–236	pCI-neo vector	AscI, PmeI
pKM6781	SpoVM-mCherry	26 residues	pCI-neo-mCherry vector (pKM 4961)	AscI
pKM4916	Cep63	1–541	pCI-neo-mCherry vector (pKM 4961)	PmeI, NotI
pKM6720	Cep63	1–541	pCI-neo-SpoVM-mCherry vector (pKM 6781)	PmeI, NotI
pKM4986	Cpe63 P1	424–541	pCI-neo-mCherry vector (pKM 4961)	PmeI, NotI
pKM6780	Cpe63 P1	424–541	pCI-neo-SpoVM-mCherry vector (pKM 6781)	PmeI, NotI
pKM6779	Cep63 P1 4A	424–541	pCI-neo-SpoVM-mCherry vector (pKM 6781)	PmeI, NotI
pKM6005	Cep152	1–1654	pEGFP-C1 (A206K) mutant vector	AgeI, SmaI
pKM6782	mGFP-Cep152 M4d	1205–1295	pcDNA3 vector	BamHI, NotI
pKM6783	mGFP-Cep152 M4d 5A	1205–1295	pcDNA3 vector	BamHI, NotI
pKM6723	mGFP-Cep152 M6	924–1295	pcDNA3 vector	BamHI, NotI
pKM6722	mGFP-Cep152 N217-M6	1–217, 924–1295	pcDNA3 vector	BamHI, NotI
pKM5767	Cep152-*sil*	1–1654	pHR′.J-CMV-SV-puro vector (pKM2994)	AscI, XhoI/ AscI, SalI
pKM5768	Cep152 5A-*sil*	1–1654	pHR′.J-CMV-SV-puro vector (pKM2994)	AscI, XhoI/ AscI, SalI
pKM6029	Cep152 6A-*sil*	1–1654	pHR′.J-CMV-SV-puro vector (pKM2994)	AscI, PmeI
pKM5659	FLAG$_3$-Cep63-*sil*	1–541	pHR′.J-CMV-SV-puro vector (pKM2994)	AscI, SalI
pKM5660	FLAG$_3$-Cep63 4A-*sil*	1–541	pHR′.J-CMV-SV-puro vector (pKM2994)	AscI, SalI

pKM6006	$FLAG_3$-Cep63 8A-*sil*	1–541	pHR′.J-CMV-SV-puro vector (pKM2994)	AscI, SalI
pKM6368	Cep152 M6	924–1295	pRSFDuet-His-mGFP (A206K)	XhoI, NotI/ SalI, NotI
pKM6459	Cep152 M6 5A	924–1295	pRSFDuet-His-mGFP (A206K)	XhoI, NotI/ SalI, NotI
pKM6066	Cep63	1–541	pETDuet-1 vector	BamHI, NotI
pKM6700	Cep63 4A	1–541	pETDuet-1 vector	BamHI, NotI
pKM6714	mGFP-Cep152 M4d	1205–1295	pETDuet-1 vector	BamHI, NotI
	mCherry-Cep63 P1	424–541		BglII, XhoI
pKM6715	mGFP-Cep152 M4d 5A	1205–1295	pETDuet-1 vector	BamHI, NotI
	mCherry-Cep63 P1 4A	424–541		BglII, XhoI
pKM6829	Cep152 N70	1–70	pKM6714	XhoI, SalI/ SalI
pKM5615	Cep63 P1	424–541	pETDuet-1 vector	BamHI, NotI
	Cep152 M4d	1205–1295		NdeI, XhoI
pKM5616	Cep63 P1 4A	424–541	pETDuet-1 vector	BamHI, NotI
	Cep152 M4d 5A	1205–1295		NdeI, XhoI
pKM5500	Cep63 P1b5	502–541	pETDuet-1 vector	BamHI, NotI
	Cep152 M4e15	1205–1257		NdeI, XhoI
pKM6018	Cep63	220–541	pETDuet-1 vector	BamHI, NotI
	Cep152 M6	924–1295		NdeI, XhoI
pKM5359	Cep63 P1b5	502–541	pETDuet-1 vector	PmeI, NotI
	Cep152 M4e15	1205–1257		NdeI, XhoI
pKM5390	Cep63 P1b5-GGGSE-Cep152 M4e10	502–541, linker, 1205–1250	pET28a-His-MBP-TEV vector	NdeI, XhoI
pKM7058	Sas6	1-490	pCI-neo-$FLAG_1$ vector (pKM2794)	SalI, NotI/ XhoI, NotI
pKM7059	Sas6 (F131E)	1-490	pCI-neo-$FLAG_1$ vector (pKM2794)	SalI, NotI/ XhoI, NotI

Supplementary Table 3. Primers used in this study

Name		Sequence (5' → 3')
Cep152	For	gtcagta ctcgag atgtcattagactttggcagtg
	Rev	gtcagta cccggg ttagtctagattaacaaatgggctatcaaagccactatc
Cep152 M4L (790-1436 aa)	For	gtcagta ctcgag ctgattgtggcagccaaactg
	Rev	gtcagta cccggg ttaggatcccacatgctttatgctc
Cep152 M4La (790–1199 aa)	For	gtcagta ctcgag ctgattgtggcagccaaactg
	Rev	gtcagta cccggg ttataaatgctgaagatgcctacagc
Cep152 M4Lb (1156–1436 aa)	For	gtcagta ctcgag ctgataagaaaaaggtccttgaaatta
	Rev	gtcagta cccggg ttaggatcccacatgctttatgctc
Cep152 5A (L1260A, I1261A, L1263A, I1266A, L1267A)	For	*gct* *gc*t tgc *gc*c caa cag *gc*t *gc*g cag gat gat gga aaa
	Rev	c*gc* a*gc* ctg ttg g*gc* gca a*gc* *agc* ata ata ttt gcg cat
Cep152 6A (L1207A, L1210A, Y1214A, I1221A, M1225A, Y1228A)	For	*gc*g gaa gaa *gc*t cgt ggg cag *gc*c att aaa gct gta aaa aaa *gc*t aaa tgt gac *gc*g ctt cgt *gc*t att cag gag agt aag
	Rev	a*gc* acg aag c*gc* gtc aca ttt a*gc* ttt ttt tac agc ttt aat g*gc* ctg ccc acg a*gc* ttc ttc c*gc* ggc tcc ccc act gca
Cep63	For	gtcagta gtttaaac atggaggctttgttagaaggaa
	Rev	gtcagta gcggccgc ctactttaaggctgtgaattgtc
Cep63 P1 (424–541 aa)	For	gtcagta gtttaaac ttacatcagcgagatatcactattg
	Rev	gtcagta gcggccgc ctactttaaggctgtgaattgtc
Cep63 P1b (490–541 aa)	For	gtcagta gtttaaac atatcttcgtgttccttgcct
	Rev	gtcagta gcggccgc ctactttaaggctgtgaattgtc
Cep63 N (1–220 aa)	For	gtcagta gtttaaac atggaggctttgttagaaggaa
	Rev	gtcagta gcggccgc ctagatagtgtcattagcccgctc
Cep63 M (210–405 aa)	For	gtcagta gtttaaac agcagtaaactggagcggg
	Rev	tcagta gcggccgc ctaactttgttcaccctgtaaaatct
Cep63 C (400–541 aa)	For	gtcagta gtttaaac cagggtgaacaaagttacagttct
	Rev	gtcagta gcggccgc ctactttaaggctgtgaattgtc
Cep63 ΔP1b (1–489 aa)	For	gtcagta gtttaaac atggaggctttgttagaaggaa
	Rev	tcagta gcggccgc ctactcatgtaaacccaattccaatt
Cep63 4A (L497A, I500A, F504A, L505A)	For	*gc*t ggt tca *gc*a gct acc aga *gc*t *gc*g gaa gag gag gaa
	Rev	c*gc* a*gc* tct ggt agc t*gc* tga acc a*gc* ggg aga tac agg
Cep63 8A (F504A, E507A, E508A, R511A, I515A, L519A, D520A, I523A)	For	*g*ct ttg gaa *gc*g *gc*g gaa ctg *gc*g tct cat cac *gc*t cta gag cgc *gc*g *gc*t gcc cat *gc*t gaa gaa cta
	Rev	a*gc* atg ggc a*gc* c*gc* gcg ctc tag a*gc* gtg atg aga c*gc* cag ttc c*gc* c*gc* ttc caa a*gc* tct ggt agc
GGGSE-Cep152 M4e-10 (1205-1250 aa)	For	gtcagta gaattc ggtggaggaagtgaa ggagccttggaagaacttcg
	Rev	gtcagta ctcgag ttattcttgacgttctcgcagta

Abbreviations: For, forward; Rev, reverse. Mutated nucleotides are shown in italic with underlines.

Supplementary Table 4. siRNA sequences used in this study

Target Gene	Sequence (nt positions from the start codon)	Source	Type
Luciferase	CGTACGCGGAATACTTCGA	9	Synthetic
CEP63	GGAGCTCATGAAACAGATT (78-96)	10	Synthetic
CEP152	GCGGATCCAACTGGAAATCTA (3099-3119)	11	Synthetic

Supplementary Table 5. Antibodies used in this study

Antibodies	Species	Cat #	Source	Exp. dilutions
Anti-FLAG (Clone M2)	Mouse	F1804	Sigma	1:1000 (IB), 1:100 (IF), 2 μg (IP)
Anti-HA (Clone 3F10)	Rat	11867423001	Roche	1:1000 (IB), 2 μg (IP)
Anti-GFP	Rabbit	sc-8334	Santa Cruz	1:1000 (IB), 1:100 (IF), 2 μg (IP)
Anti-mCherry	Rabbit	26765-1-AP	Proteintech	1:1000 (IB)
Anti-Cep63 (Full-length)	Rabbit	06-1292	EMD Millipore	1:1000 (IB), 1:100 (IF)
Anti-Cep152 (N-terminal; GT1285)	Mouse	GTX631486	GeneTex	1:100 (IF)
Anti-Cep152 M (491-810)	Rabbit	Lab supply	50	1:1000 (IB), 1:100 (IF)
Anti-Plk4 (580-970)	Rabbit	Lab supply	50	1:50 (IF)
Anti-SAS6 (404-657)	Mouse	sc-81431	Santa Cruz	1:100 (IF)
Anti-Cep192 (1-647)	Rabbit	Lab supply	50	1:100 (IF)

Fig. 1a

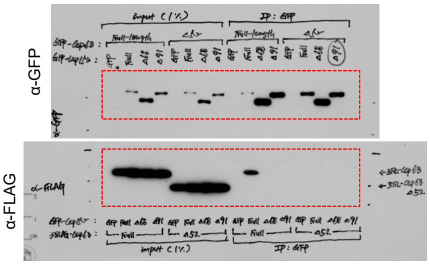

Fig. 1b

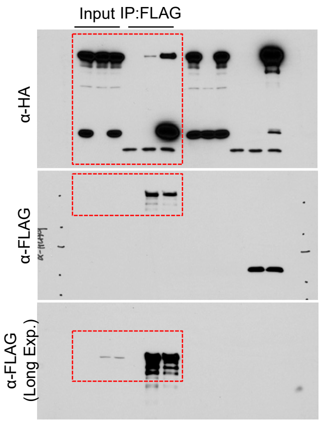

Fig. 1c

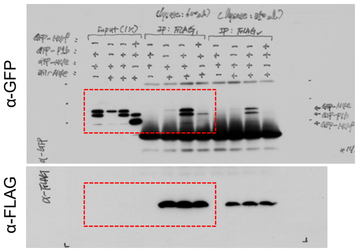

Fig. 3e

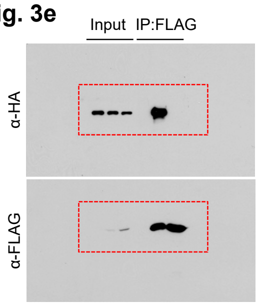

Fig. 3f

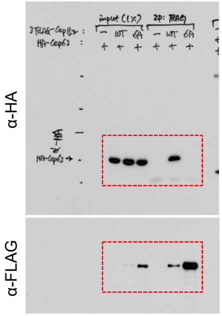

Fig. 3g

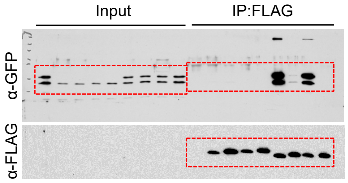

Fig. 4b

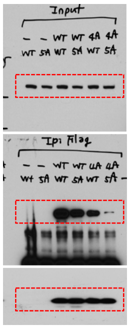

Fig. S1a

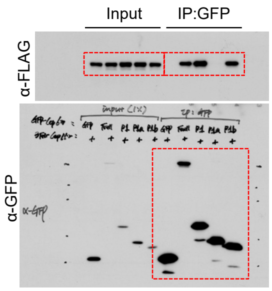

Fig. S1b

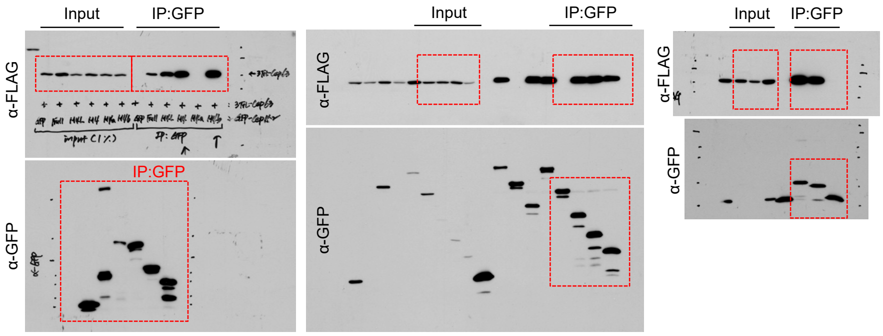

Fig. S1e

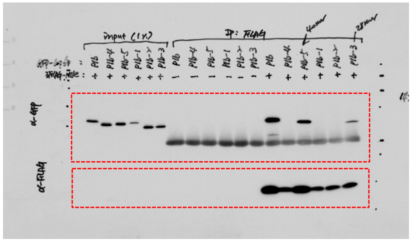

Fig. S1f

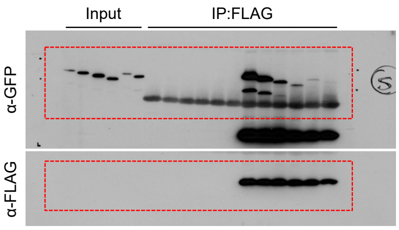

Fig. S1g

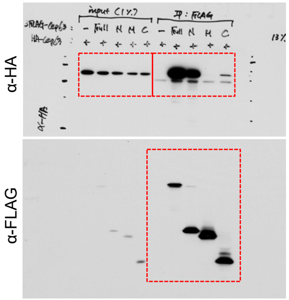

Fig. S1h

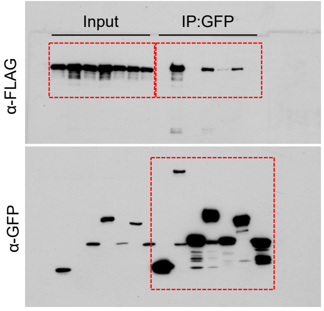

Fig. S1i

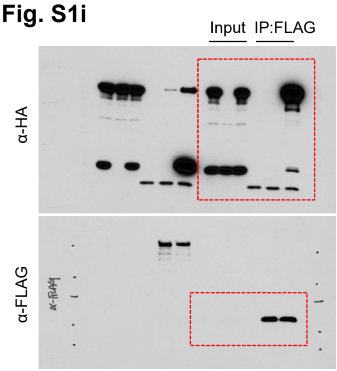

Fig. S1j

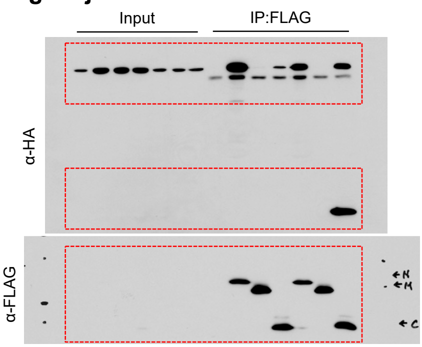

Fig. S1l

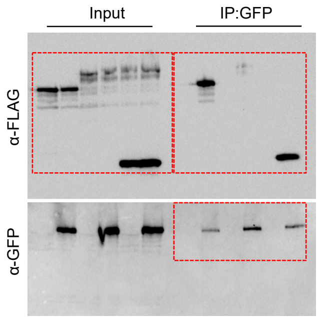

Fig. S3h

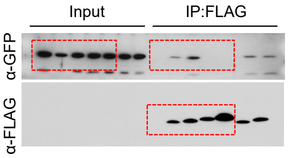

Fig. S3i

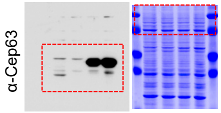

Fig. S3l

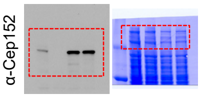

Fig. S4a

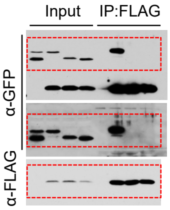

Fig. S4b

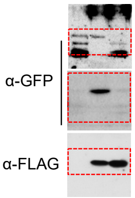

Fig. S4d

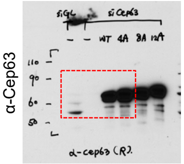

Fig. S4g

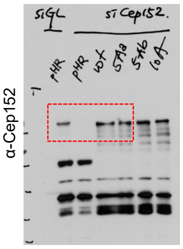

Fig. S5a

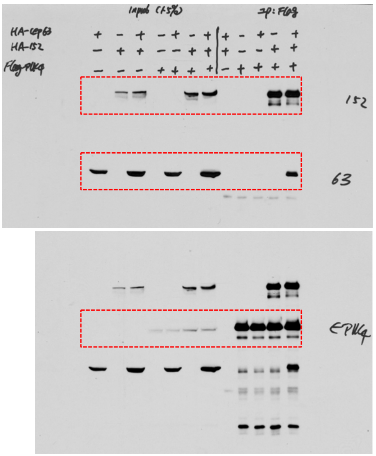

Fig. S5b

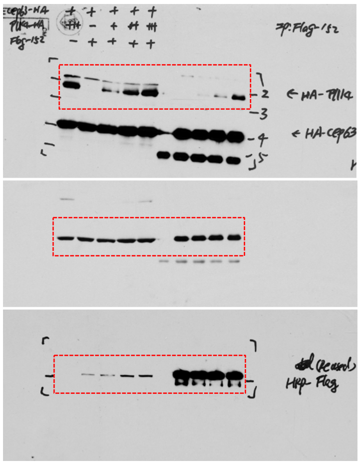

Fig. S7d

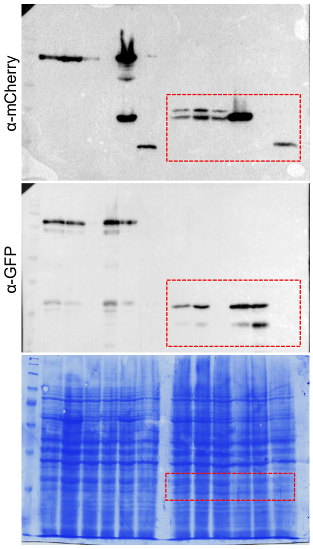

Supplement: Supplementary file 1 — Supplementary Information [file 41467_2019_8838_MOESM1_ESM.pdf]
