# Supplementary material for: Molecular architecture of a cylindrical self-assembly at human centrosomes
Source: Nat Commun. 2019 Mar 11;10:1151. doi: 10.1038/s41467-019-08838-2 (PMC6411776; doi:10.1038/s41467-019-08838-2)
Supplement: Supplementary file 9 — Description of Additional Supplementary Files [file 41467_2019_8838_MOESM9_ESM.docx]

**Title:** Supplementary Movie 1.
**Description:** 3-D reconstruction of images shown in Fig. 2c top.

**Title:** Supplementary Movie 2.
**Description:**  3-D reconstruction of images shown in Fig. 2c bottom.

**Title:** Supplementary Movie 3.
**Description:** Time-lapse video of the data shown in Fig. 2h. Bar, 500 nm. Video starts 30 minutes after placing the mCherry-Cep63 P1•mGFP-Cep152 M4d complex on a coverslip for SIM-TIRF at RT.

**Title:** Supplementary Movie 4.
**Description:** 3-D reconstruction of images shown in Fig. 5a.

**Title:** Supplementary Movie 5.
**Description:** 3-D reconstruction of the assembly shown in Fig. 5b bottom.

**Title:** Supplementary Movie 6.
**Description:** 3-D reconstruction of images shown in Supplementary Fig. 2f.

**Title:** Supplementary Movie 7.
**Description:** 3-D reconstruction of images shown in Supplementary Fig. 7e.
